# Parameterization of high magnetic field gradient fractionation columns for applications with *Plasmodium falciparum *infected human erythrocytes

**DOI:** 10.1186/1475-2875-9-116

**Published:** 2010-05-03

**Authors:** Stephan Karl, Timothy ME Davis, Tim G St Pierre

**Affiliations:** 1 School of Physics, M013, The University of Western Australia, 35 Stirling Highway, Crawley WA 6009, Australia; 2 School of Medicine and Pharmacology, The University of Western Australia, Fremantle Hospital, Alma Street, Fremantle, WA 6959, Australia

## Abstract

**Background:**

Magnetic fractionation of erythrocytes infected with *Plasmodium falicparum *has several research uses including enrichment of infected cells from parasite cultures or enhanced detection of *P. falciparum *gametocytes. The aim of the present study was to quantitatively characterize the magnetic fractionation process and thus enable optimization of protocols developed for specific uses.

**Methods:**

Synchronized cultures of *P. falciparum *parasites incubated with human erythrocytes were magnetically fractionated with commercially available columns. The timing of the fractionation experiments was such that the parasites were in second half of their erythrocytic life cycle with parasite densities ranging from 1 to 9%. Fractionations were carried out in a single pass through the columns. Cells were enumerated and differentiated in the initial samples as well as in the positive and negative fractions. The capture of cells by the fractionation column was described by a saturation binding model.

**Results:**

The magnetic binding affinity to the column matrix was approximately 350 times greater for infected cells compared with uninfected cells. The purity of infected cells in the captured fraction was generally >80% but decreased rapidly (to less than 50%) when the number of infected cells that passed through the column was substantially decreased (to less than 9 ± 5 × 10^5 ^cells). The distribution of captured parasite developmental stages shifted to mature stages as the number of infected cells in the initial samples and flow rate increased. The relationship between the yield of infected cells in the captured fraction and flow rate of cells conformed to a complementary cumulative log-normal equation with flow rates >1.6 × 10^5 ^cells per second resulting in yields <50%.

**Conclusions:**

A detailed quantitative analysis of a batchwise magnetic fractionation process for malaria infected erythrocytes using high gradient magnetic fractionation columns was performed. The models applied in this study allow the prediction of capture efficiency if the initial infected cell concentration and the flow rate are known.

## Background

*Plasmodium *species have complex life cycles involving the invertebrate mosquito and human host [1]. In humans, the parasite undergoes asexual multiplication in red blood cells where haemoglobin provides the main source of the protein necessary for its development. Haemoglobin is transported in aliquots to the parasitic lysosome and digested [2]. Haem groups are by-products of this process. The iron in the haem rapidly oxidizes and haem monomers are converted into an inert crystalline and paramagnetic material named haemozoin or malaria pigment which accumulates in infected erythrocytes [3,4]. The rate of haemozoin formation correlates with parasite metabolic activity and peaks at the trophozoite stage of development [5].

Magnetic fields can be used to separate *Plasmodium*-infected blood samples into positive and negative fractions with a higher and lower percentage of infected cells, respectively, than the initial sample [6,7]. The most commonly used magnetic cell fractionation system, including that for malaria parasites, is the MACS system manufactured by Miltenyi Biotec (Bergisch-Gladbach, Germany). The MACS system utilizes columns containing a matrix of magnetic beads as magnetic field gradient enhancing medium. When placed into an external magnetic field, the beads become magnetized. Strong local magnetic fields and field gradients facilitate binding of cells with magnetic susceptibilities sufficiently different from their surrounding medium. These cells can be eluted from the columns when the external magnetic field is removed.

Apart from its usage for parasite synchronization [8,9] and *in-vitro *biochemical [10], biophysical [11], molecular [9,12] and immunological studies [13,14], magnetic fractionation has found its application in the isolation of rare parasitized cells from peripheral blood of malaria infected patients [13,15,16].

In general, there are two classes of *Plasmodium falciparum *infected cells, to which magnetic fractionation is applicable and which may occur in very low concentrations in peripheral blood. Firstly, asexual mature stages of *P. falciparum*, which express proteins that facilitate cytoadherence to the vascular endothelium [17,18]. Apart from in severe malaria infections, these stages are very rarely observed on blood smears because the vast majority is sequestered. Magnetic fractionation has been used to isolate these mature asexual stages [15,16].

Secondly, magnetic fractionation can be used to improve detection and quantification of gametocytes from peripheral blood [15,16,19,20]. Gametocytes, the sexual stages of *Plasmodium *that are taken up by the mosquito host, also contain haemozoin crystals but do not cytoadhere in their final stages of development. They are typically much less numerous than asexual parasite stages and their prevalence is often underestimated [21,22].

Magnetic fractionation can be applied to the other *Plasmodium *species that infect humans [16,20]. *Plasmodium vivax, Plasmodium malariae *and *Plasmodium ovale *do not cytoadhere, and late trophozoite and schizont stages of these species are found more frequently in peripheral blood [23,24]. However, laboratory studies are usually confined to *P. falciparum *since continuous culture of the other species is not currently feasible.

Although different customized magnetic fractionators have been applied, most studies have used the commercially available MACS system.

There has been no quantitative analysis of the magnetic fractionation process applied to malaria infected cell suspensions. Such data are required to establish conditions for optimal purity and yield such as when infected cell densities are too low to be detected by conventional light microscopy. In the present study this analysis was performed using cultured *P. falciparum *and the widely-available MACS equipment.

## Methods

### Continuous parasite culture

The laboratory-adapted *Plasmodium falciparum *strains 3D7 and W2mef were cultured in RPMI 1640 HEPES (Sigma Aldrich, St Louis, MO) supplemented with 92.6 mg/L L-glutamine (Sigma Aldrich, St Louis, MO), 500 mg/L gentamicin (Sigma Aldrich, St Louis, MO), 50 mg/L hypoxanthine (Sigma Aldrich, St Louis, MO) and 10% v/v pooled human plasma. Cultures were maintained with daily changes of culture medium at 5% haematocrit and diluted with red blood cells when parasitaemia exceeded 5%. Cultures were incubated in an airtight desiccator cabinet at 37°C in an atmosphere containing between 5% to 10% oxygen. The low oxygen atmosphere was generated by gassing the cabinet with a mixture of 1% O_2 _and 5% CO_2 _balanced in N_2 _(BOC gases, Perth, Australia) at 1.0-1.5 bar for 60-90 seconds each time it had been opened.

### Synchronization

The accumulation of haemozoin is a gradual process over the 48 h of the intraerythrocytic life cycle of *Plasmodium falciparum*. The conversion rate is maximal at the trophozoite stage, as are many of the parasite's metabolic processes. The assays described below required parasites at the same stage of development after merozoite invasion. This synchronization was achieved by suspension of the cells in 5% w/v sorbitol (Sigma Aldrich, St Louis, MO) for 12 min to allow destruction of mature parasite stages through osmotic pressure change, followed by re-suspension of erythrocytes containing viable parasite forms in culture medium. Such single-step sorbitol synchronization produces cultures that contain parasites, which are ring and early trophozoite stages that have developed for an estimated maximum time of 18 h after merozoite invasion.

### Magnetic fractionation

The present study comprised a total of 45 magnetic fractionation experiments. Of these, 30 were conducted with varying initial cell concentrations (15 experiments with the chloroquine and mefloquine sensitive strain 3D7, and 15 with chloroquine and mefloquine resistant strain W2mef) and 15 experiments with varying flow rates (10 with 3D7 and 5 with W2mef).

Magnetic fractionations were conducted 24 h after synchronization, so that parasites were in the second half of the life cycle (24 h-42 h). A MidiMACS magnet, a MACS-multistand and LS columns (Miltenyi Biotech, Bergisch Gladbach, Germany) were used for the experiments. The columns were placed into the magnet unit (which generates a magnetic field of approximately 0.65 T) and primed with 0.7 mL sterile filtered magnetic fractionation buffer (MFB, PBS pH 7.4, containing 0.5 g/L bovine serum albumin and 0.0037 g/L EDTA) equilibrated to room temperature. Before passage over the column, each sample of erythrocytes was washed once in PBS, spun down, and then resuspended in 5 mL MFB in 15-mL centrifuge tubes (BD Biosciences, San Jose, CA) and incubated at room temperature for at least 10 min.

Different flow rates were achieved by attachment of sterile syringe needles (BD Biosciences, San Jose, CA) with different internal diameters and lengths to the end of the MACS columns. The volumetric flow rate was determined by measuring the time it took for a sample of defined volume to pass through the column. The effluent (hereafter termed the negative fraction) was collected in 50 mL cone tubes (BD Biosciences, San Jose, CA).

The columns were then washed with 2 × 1 mL MFB and removed from the magnet. The sterile needle was disconnected and the cells captured in the column (hereafter termed the positive fraction) were eluted into 15-mL centrifuge tubes (BD Biosciences, San Jose, CA) by washing with 1 × 5 mL MFB.

### Measurements of cell concentrations

All 15 mL and 50 mL cone tubes were weighed before and after each experiment to determine the volumes of the cell suspension in the initial samples, and the positive and negative fractions. The density of the fractionation buffer was determined by weighing a 1 mL volume measured using a calibrated pipette on a precision laboratory scale multiple times (n = 6). The density of the magnetic fractionation buffer was 0.994 ± 0.02 g/mL.

A volume of 200 μL of cell suspension was removed from both the initial sample and the negative fraction and measured with a cell counter (Cell Dyn 4000, Abbott, Lane Cove, NSW, Australia) which reliably measures erythrocyte concentrations above a threshold of 2 × 10^3 ^cells per mL. The initial and negative fractions contained an average of 1.16 × 10^8 ^cells per mL (range: 4.97 × 10^5 ^cells/mL to 7.62 × 10^8 ^cells/mL), which is well above the required threshold of the instrument.

Because the cell concentrations in the positive fractions can be much smaller, a haemocytometer (Neubauer type, Brand GmbH + Co KG, Wertheim, Germany) was used to determine the cell concentrations in the positive fractions. An aliquot of 10 μL cell suspension was introduced into the counting chamber of the haemocytometer and the red blood cell concentration determined by counting the number of red blood cells in 4 large squares (0.4 mm^3^) of the counting chamber.

### Flow cytometry

Since only parasitized erythrocytes contain DNA, fluorescence staining of the DNA and subsequent flow cytometry can be used to reliably discriminate between infected and uninfected erythrocytes and to determine total parasitaemia [25-27]. Aliquots of 1 μL of packed cells from the initial and the negative fractions, and from 1 mL of the positive fractions, were suspended in 1 mL PBS (pH 7.4) and the double-stranded DNA was stained by addition of 1 μL Sybr Green nucleic acid gel stain (10,000× concentrate in DMSO, Invitrogen Molecular Probes, Mulgrave, Australia). The samples were incubated in the dark at 37°C for 15 min. Excess stain was removed by washing once in PBS (pH 7.4). The samples were then fixed in a PBS (pH 7.4) solution containing 2.5% v/v glutaraldeyde and 1% v/v paraformaldehyde for 60 minutes at 4°C. The fixative solution was replaced by PBS (pH 7.4) and the samples were stored at 4°C in the dark until analysis on a FACSCanto II flow cytometer (BD Biosciences, San Jose, CA). The flow cell in this instrument was a quartz cuvette and the light source a 20 mW/488 nm solid state argon laser. At the flow cell, the effective power of this light source is typically 15 mW. The laser beam had an elliptical geometry with 9 mm and 65 mm being the short and long axes of the beam profile respectively. The instrument was equipped with photomultiplier tube detectors to detect wavelengths emitted from interaction with the 488 nm argon laser in the ranges of 750-810 nm, 670-735 nm, 610-637 nm and 564-606 nm. Sybr Green has its emission maximum at 520 nm and therefore the 564-606 nm channel was selected from those available on the instrument for detection. Fifty thousand events were acquired from each sample.

### Differential parasite count

**Figure 1:**
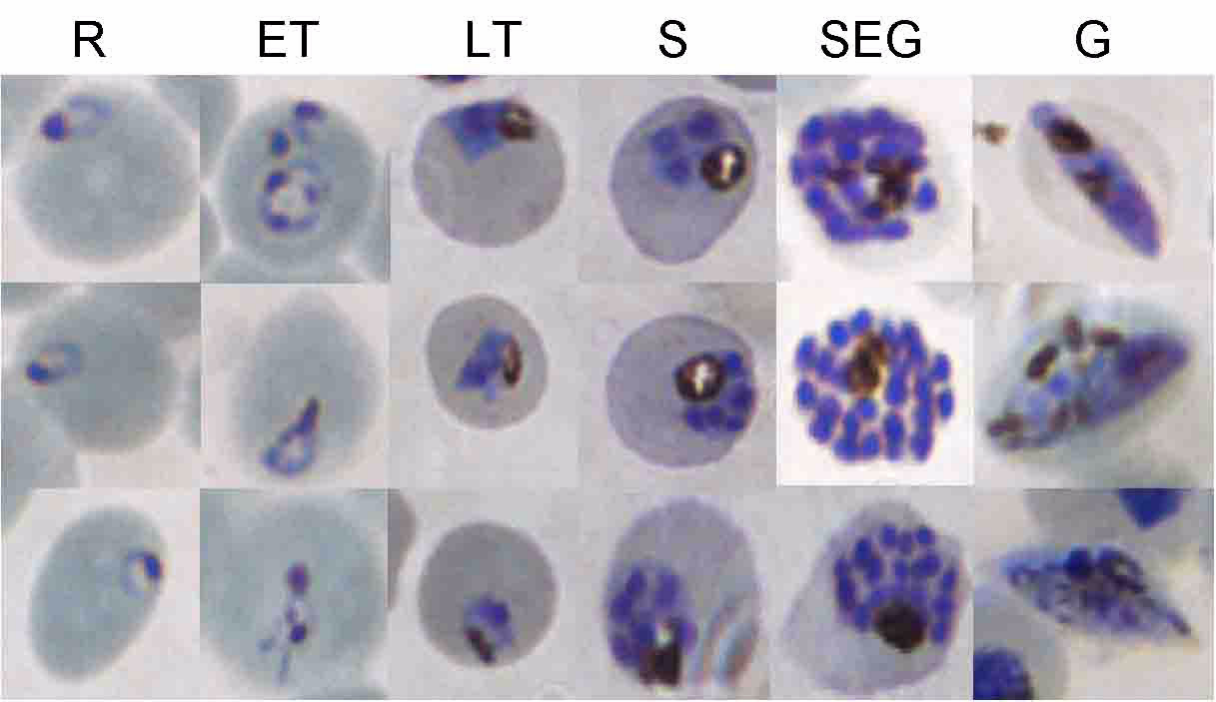
**Exemplary images of infected erythrocytes at different stages of infection**. Three examples of each of the different intraerythrocytic developmental stages of *Plasmodium falciparum *reflecting the classification used in the present study. An early trophozoite (ET) was distinguished from a ring stage (R) when the cytoplasm of the parasite had begun to expand but no haemozoin crystals were visible. A late trophozoite (LT) contained haemozoin and not more than one nucleus. A schizont (S) contained up to 8 nuclei. Parasites with >8 nuclei were classified as segmenters (SEG). Examples of gametocytes (G) are also shown. Images were obtained on a Nikon Eclipse TE2000 -N Microscope with a 1000× optical magnification with a Nikon LH-M100CB-1 Camera.

Thin blood smears were prepared from each initial sample and from the positive and negative fractions in each experiment. Parasite development was divided into 6 stages: Rings (R), early trophozoites (ET), late trophozoites (LT), schizonts (S), segmenters (SEG) and gametocytes (G). Photomicrographs depicting parasites in the different developmental stages are shown in Figure 1. One hundred parasites were counted and staged on each blood smear.

## Results

**Figure 2:**
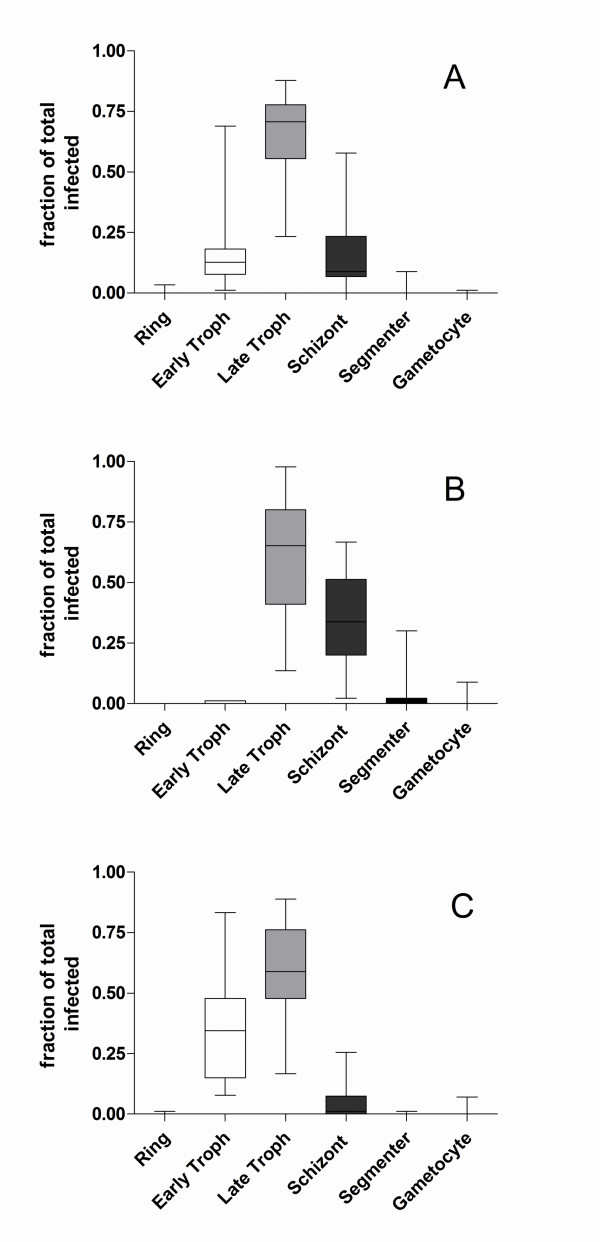
**Distribution of parasite stages before and after magnetic fractionation**. Distribution of parasitic developmental stages in the initial samples (panel A), the positive fractions (panel B) and the negative fractions (panel C) for all 45 experiments conducted in the present study. The boxes denote the interquartile ranges, the whiskers denote the ranges, and the horizontal lines in the boxes denote the medians of each group.

The distributions of parasite stages in the initial samples as well as in the positive and negative fractions are shown in Figure 2.

The late trophozoite stage was the most prevalent stage in all fractions. Few early trophozoites and rings were seen in the positive fractions whereas schizonts, segmenters and gametocytes were infrequent in the negative fractions. Rings and gametocytes together accounted for <1% of the total parasite population in each of the fractions. The average total recovery of infected cells in the positive fractions in all 45 experiments was 88% (95% confidence interval: 81%-95%). The parasitaemia in the intial samples ranged from 0.92% to 8.71%, from 56.4% to 96.7% in the positive fractions and from 0.51% to 3.34% in the negative fractions.

**Figure 3:**
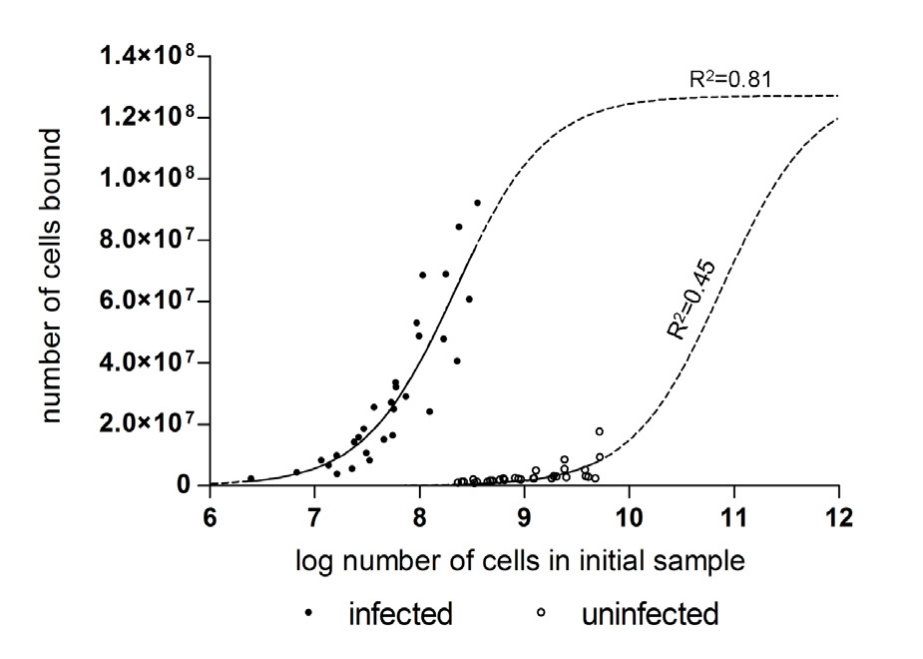
**Binding characteristics of infected cells and uninfected cells to MACS LS columns**. The lines of best fit were obtained by fitting equation (1) to the data.

Figure 3 shows the numbers of infected and uninfected cells that bound to the column against the number in the initial sample.

A simple saturation binding model (Equation 1 below) which estimates the number of available binding sites in the column and compares the relative affinities of infected and uninfected cells for the bead matrix was fitted independently to the two sets of data:(1)

where *B*_max _is the effective number of available binding sites in the column, *N*_*I *_is the number of cells in the initial fraction and *N*_*P *_is the number of cells in the positive fraction. The model takes no account of competition between the infected and uninfected cells for binding sites. This simplification can be justified on the basis that, in all experimental situations, the number of uninfected cells that bind to the column will always be much lower than the number of available binding sites.

When applied to the infected cells, the model yielded an estimate of the number of potential binding sites in the column of 1.27 (± 0.27) × 10^8^. This number approximates the number of cells in 13 μL of packed red blood cells (at 10^7 ^cells per μL packed cells). The half saturation constant *K*_*D *_for infected cells was 2.18 (± 0.95) × 10^8^. The predicted maximum number of uninfected cells was very similar to the number of infected cells (1.29 × 10^8^). However the curve fit for the uninfected cells was suboptimal since the applied uninfected cell densities are all much lower than the predicted *K*_*D *_for uninfected cells of 7.7 × 10^10^. It should be noted that the *K*_*D *_for uninfected cells corresponds to 7.7 mL packed red blood cells and that application of cell volumes of this magnitude to the columns is not feasible. Based on the ratio of the *K*_*D *_values it can be estimated that the column binding affinity of infected cells (with the distribution of parasite developmental stages described earlier) is about 350 times higher than that for uninfected cells.

**Figure 4:**
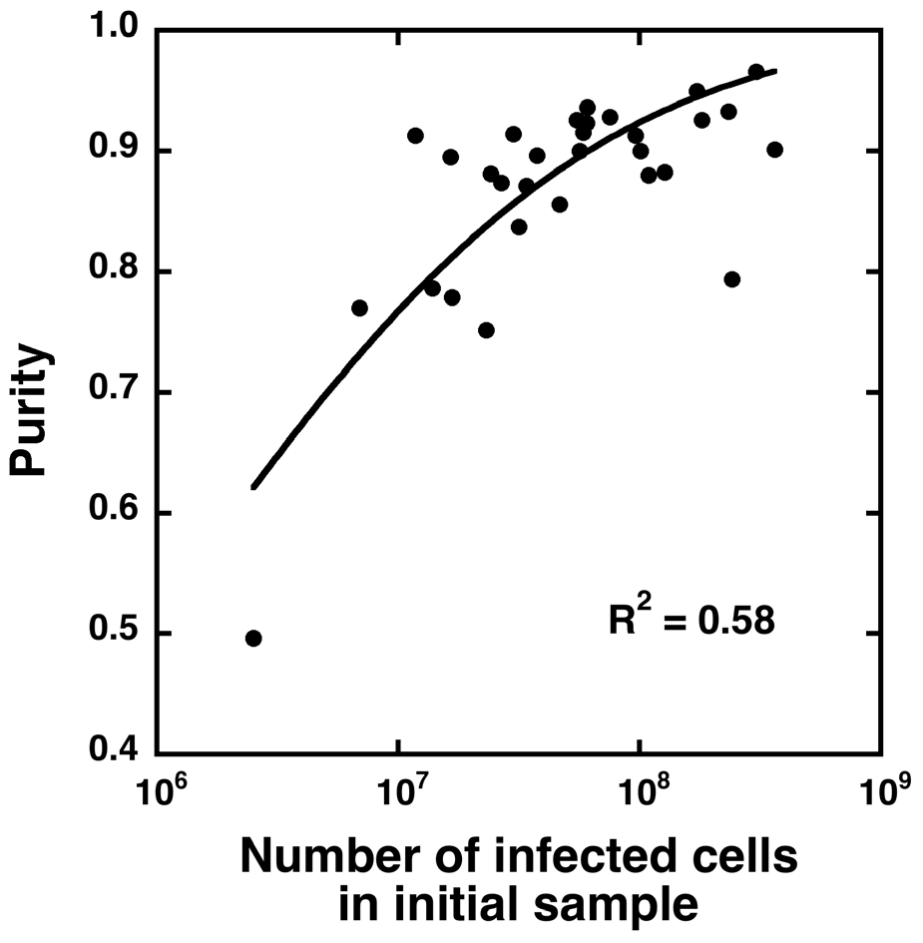
**Purity of infected cells in the positive fraction against number of infected cells in the initial sample**. The solid line is a fit of equation (2) to the data.

Despite the initial assumptions inherent in the model, there was evidence for competition for binding between infected and uninfected cells at very low infected cell densities. Since the binding affinity of the uninfected cells is low, a standard competition binding analysis could not be conducted. However, Figure 4 shows a decrease in the purity of the infected cells in the positive fraction as the number of infected cells in the initial sample is substantially decreased, indicating competition for binding sites. The data in Figure 4 were fitted to a cumulative log normal distribution of the form:(2)

where *n*_*i *_is the number of infected cells in the initial sample and *n*_50% _and *σ*_*purity *_are fitted parameters and the complementary error function is given by

The parameter *n*_50% _was found to be 9 (±5) × 10^5 ^and is the number of infected cells in the initial sample below which the equation predicts that the purity in the positive fraction will be less than 50%. The parameter *σ*_*purity *_was 3.3 ± 0.5.

**Figure 5:**
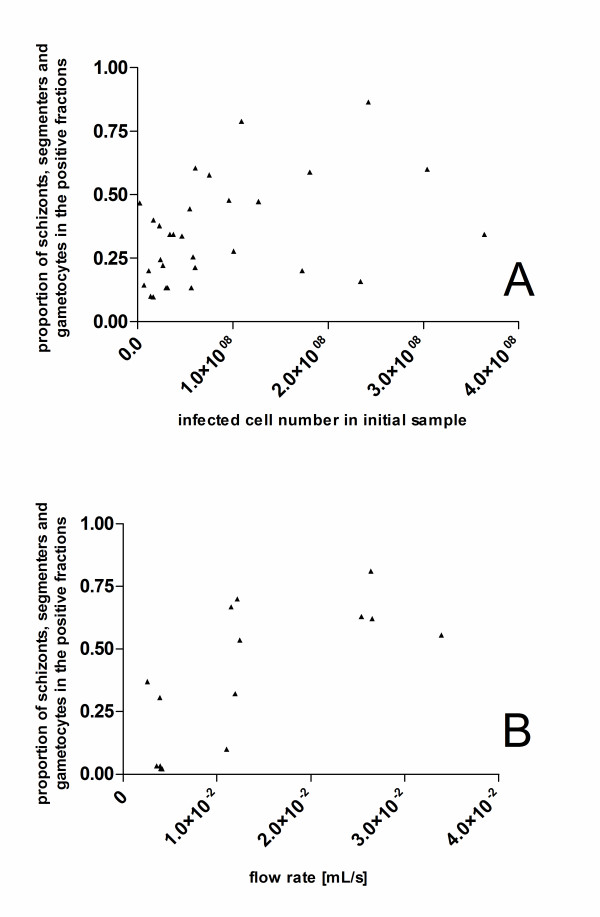
**Influence of flow rate and initial cell number on the stage distribution in the positive fractions**. Shift in the proportion of infected cells in the positive fractions that are schizonts, segmenters or gametocytes with changing initial infected cell number (panel A, Spearman correlation coefficient: + 0.48, p = 0.0068, n = 30) and flow rate (panel B, Spearman correlation coefficient: + 0.67, p = 0.0064, n = 15). Stages containing higher quantities of haemozoin bind preferentially to the bead matrix at higher initial infected cell numbers and higher flow rates.

With increasing numbers of infected cells in the initial sample, there was a significant increase in the proportion of haemozoin-containing infected cells (late trophozoites, schizonts, segmenters and gametocytes) in the negative fractions (Spearman correlation coefficient 0.37, p = 0.04, n = 30). This positive association is most likely related to column saturation since at the higher initial numbers of infected cells the capacity of the columns is exceeded by approximately 3-fold resulting in approximately 75% of the binding sites being occupied (see Figure 3). Hence, there is a higher probability of haemozoin-containing cells passing through the column without binding at these higher initial infected cell numbers. Furthermore, the proportion of infected cells in the positive fraction in the form of schizonts, segmenters and gametocytes increased with higher initial cell concentrations (Figure 5A). A similar but more marked increase in the proportion of infected cells in the positive fraction in the form of schizonts and segmenters was found with increasing flow rate (Figure 5B). Both phenomena can be explained by the stronger magnetic force exerted on schizonts and segmenters. These stages contain higher quantities of haemozoin and thus bind preferentially to the bead matrix.

**Figure 6:**
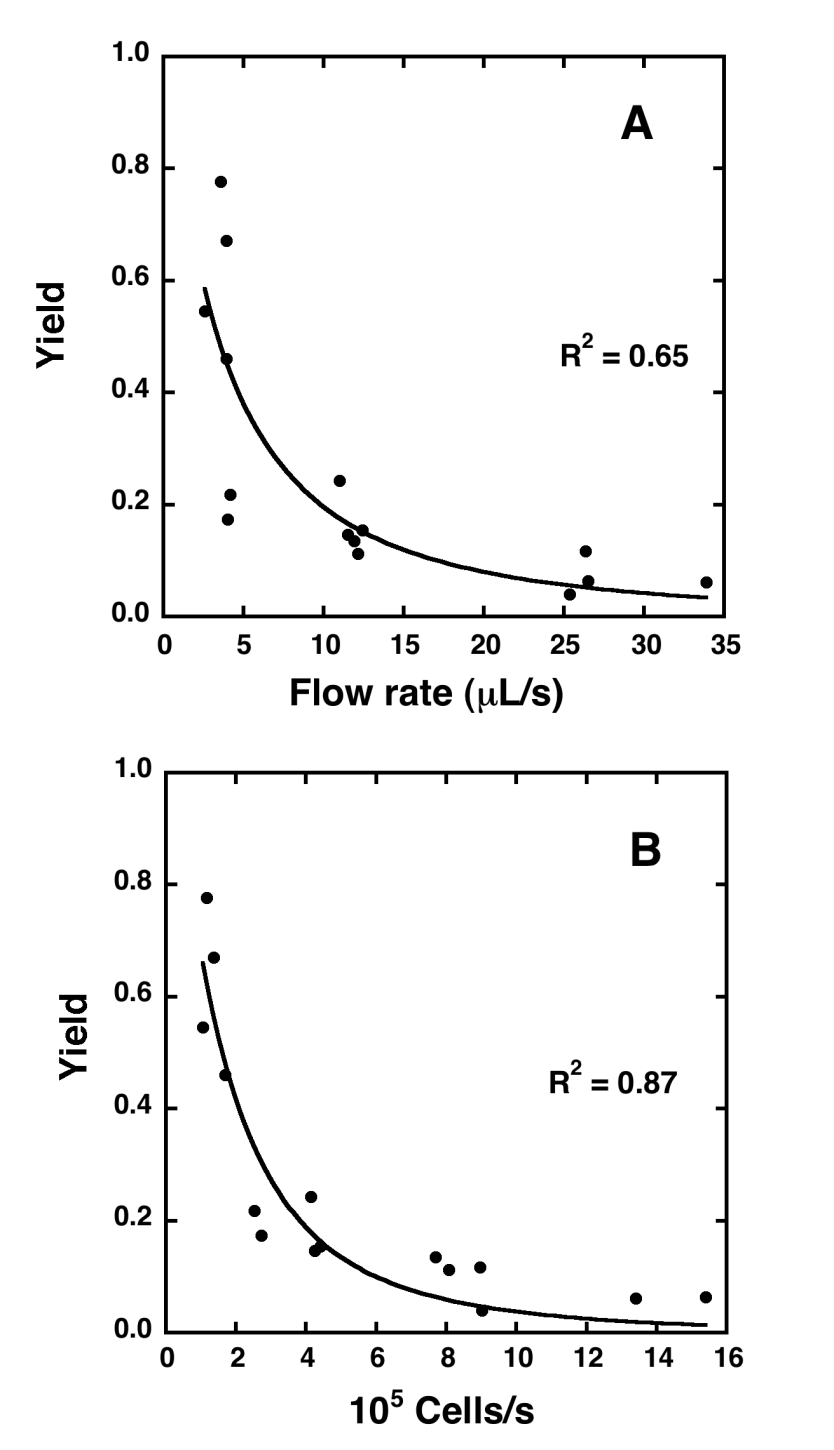
**Dependence on the infected cell yield in the positive fractions on flow rate and initial cell concentration**. Dependence of the yield of infected cells in the positive fraction (A) on flow rate through the column and (B) on the product of flow rate and concentration of infected cells in the initial sample. The solid lines are fits of equations (3) and (4) to the data.

The flow rate had a strong influence on the number of infected cells retained in the fractionation columns. With increasing flow rate, the infected cell yield in the positive fraction decreased monotonically (Figure 6A). A complementary cumulative log-normal function,(3)

was fitted to the data where F is the flow rate through the column. The fit yielded the following parameters with a coefficient of determination R^2 ^= 0.65: *μ*_1 _= -5.7 ± 0.2 and *σ*_1 _= 1.3 ± 0.3. A flow rate of *e*^*μ*^^1 ^(= 0.0034) mL/s corresponds to that at which the equation predicts the yield to be 50%. The product of flow rate and concentration of infected cells in the initial sample (*F *× *c*_*i*_) correlated more strongly with the infected cell yield than flow rate alone (Figure 6B). When the following equation was fitted to the data in Figure 6B,(4)

an improved coefficient of determination (R^2 ^= 0.87) was obtained together with values of *μ*_2 _and *σ*_2 _of 12.0 ± 0.1 and 1.03 ± 0.15 respectively. The fitted equation predicts an infected cell yield of 50% when the product of flow rate and concentration of infected cells in the initial sample is *e*^*μ*^^2 ^(= 1.6 × 10^5 ^cells/s).

There was no relationship between the purity of the positive fractions and flow rate. The average purity in the positive fractions in the flow rate experiments (n = 15) was 83.5 ± 10.5%.

There were no statistically significant differences in any of the derived parameters between the two different strains of *P. falciparum *used in the present study.

## Discussion

Many studies have applied magnetic fractionation in malaria research but only few have made any attempt to quantify the fractionation results. A common theme in all previous studies of the actual magnetic fractionation process [8,15,16,19,20] is the generation of qualitative information on 'enrichment' (fold increase), 'concentration' or 'purification' based on blood smear microscopy. However, assessing purity in the positive fractions based on slide counts alone can be misleading and does not provide information on capture efficiency and the distribution of parasitized cells recovered in the different fractions. The present study shows clearly that purity can be influenced by a number of different factors related to the nature of the parasite, the blood sample and its infected red cell density. In addition, some *P. falciparum *strains may bind to uninfected cells to form rosettes [28,29], a process that could theoretically lead to major losses in purity despite the use of anticoagulants and possible mechanical disruption of rosettes during sample processing. Furthermore, purity in the positive fraction is dependent on the volume of buffer applied to elute unbound cells. While larger buffer volumes might increase the purity, this strategy will decrease the numbers of infected cells recovered in the positive fraction, since the infected cells are not tightly bound to the column matrix and will be eluted with uninfected cells.

Iron in methaemoglobin exhibits paramagnetic characteristics similar to those of haemozoin iron. In healthy erythrocytes, the amount of methaemoglobin is usually <1% of the total haemoglobin but it may exceed 8-10% in methaemoglobinaemia [30]. Malaria induced methemoglobinemia has been reported but is related to free methemoglobin generated due to the increased rupture of healthy erythrocytes during malaria infection [31,32]. Only intra-erythrocytic elevation of methemoglobin levels could lead to a significant retention of uninfected cells in magnetic fractionation columns. The present study shows that the binding kinetics strongly favour infected cells which have an affinity that is two orders of magnitude higher than that of uninfected cells.

There were no parasite strain-specific differences in found in the present data. However, it should be noted that different parasite strains may vary slightly in their growth characteristics over a single life cycle. This could theoretically lead to small differences in the amount of haemozoin present in infected cells 24 h after synchronization [33], the time point at which the present experiments were conducted.

While flow rate did not influence purity, there was a monotonic decrease in infected cell yield for all stages of infection as flow rate increased. This suggests that haemozoin containing cells are not strongly retained in the MACS columns. The magnetic fractionation process may be more comparable with column chromatography, where the retention time of infected cells in the columns is a function of haemozoin content. The present study shows that the highest capture efficiencies in the magnetic fractionation columns are achieved at a minimal flow rate and minimal concentration of infected cells. The highest capture efficiencies achieved in this study were >75% infected cells. While the number of uninfected cells in the initial sample did have an effect on infected cell purity in the positive fractions, it did not influence capture efficiency significantly. Therefore, in a clinical setting where only a few haemozoin containing cells circulate in peripheral blood, the probability of capturing infected cells can be increased by lowering the flow rate and increasing the sample volume without diminishing capture efficiency. The models applied in this study allow the prediction of capture efficiency if the initial infected cell concentration and the flow rate are known.

## Conclusion

The present study is the most detailed quantitative characterization of magnetic fractionation of erythrocytes infected with *Plasmodium falciparum *conducted to date. Although the present analysis was confined to the use of one kind of separation column and specific parasite strains, the present approach can be used to guide quantitation of any batch-wise magnetic cell fractionation process used in malaria-infected erythrocytes. If magnetic fractionation is used for concentration of microscopically undetectable numbers of haemozoin-containing infected cells (e.g. from patient blood), the protocol presented here provides the means to estimate the density of these cells in the initial sample from a blood smear prepared from the positive fraction.

## List of Abbreviations

95% CI: 95% confidence interval; DMSO: dimethyl sulfoxide; DNA: desoxyribonucleic acid; EDTA: ethylenediaminetetraacetic acid; ET: Early trophozoite; G: gametocyte; HEPES: 4-(2-hydroxyethyl)-1-piperazineethanesulfonic acid; LT: late trophozoite; MACS: magnetically activated cell sorting; MFB: magnetic fractionation buffer; PBS: phosphate buffered saline; R: ring stage; RPMI: Ross Park Memorial Institute; S: schizont; SEG: segmenter.

## Competing interests

The authors declare that they have no competing interests.

## Authors' contributions

SK, TMED and TSP conceived the study, SK performed the laboratory work, SK, TMED and TSP wrote the manuscript. All authors read and approved the final manuscript.
